# Anti-CD3 Antibody Treatment Reduces Scar Formation in a Rat Model of Myocardial Infarction

**DOI:** 10.3390/cells9020295

**Published:** 2020-01-25

**Authors:** Bernhard Wernly, Vera Paar, Achim Aigner, Patrick M Pilz, Bruno K Podesser, Martin Förster, Christian Jung, Josefina Pinon Hofbauer, Birgit Tockner, Monika Wimmer, Theo Kraus, Lukas J Motloch, Matthias Hackl, Uta C Hoppe, Attila Kiss, Michael Lichtenauer

**Affiliations:** 1 Department of Internal Medicine II, Division of Cardiology, Paracelsus Medical University Salzburg, 5020 Salzburg, Austria; bernhard_wernly@airpost.net (B.W.); v.paar@salk.at (V.P.); l.motloch@salk.at (L.J.M.); u.hoppe@salk.at (U.C.H.); 2 Division of Cardiology, Department of Medicine, Karolinska Institutet, Karolinska University Hospital, 171 77 Stockholm, Sweden; 3 Rudolf Boehm-Institute for Pharmacology and Toxicology, Clinical Pharmacology, Leipzig University, Faculty of Medicine, 04107 Leipzig, Germany; Achim.Aigner@medizin.uni-leipzig.de; 4 Ludwig Boltzmann Institute for Cardiovascular Research at the Center for Biomedical Research, Medical University Vienna, 1080 Vienna, Austria; patrick.pilz@meduniwien.ac.at (P.M.P.); bruno.podesser@meduniwien.ac.at (B.K.P.); attila.kiss@meduniwien.ac.at (A.K.); 5 Universitätsherzzentrum Thüringen, Clinic of Internal Medicine I, Department of Cardiology, Friedrich Schiller University Jena, 07743 Jena, Germany; Martin.Foerster@med.uni-jena.de; 6 Division of Cardiology, Pulmonology, and Vascular Medicine University Duesseldorf, Medical Faculty, 40225 Duesseldorf, Germany; Christian.Jung@med.uni-duesseldorf.de; 7 EB House Austria, Research Program for Molecular Therapy of Genodermatoses, Department of Dermatology and Allergology, University Hospital of the Paracelsus Medical University Salzburg, 5020 Salzburg, Austria; j.d.pinon@salk.at (J.P.H.); b.tockner@salk.at (B.T.);; 8 Department of Pathology, Paracelsus Medical University Salzburg, 5020 Salzburg, Austria; t.kraus@salk.at; 9 TAmiRNA GmbH, Leberstrasse 20, 1110 Vienna, Austria; matthias.hackl@tamirna.com

**Keywords:** myocardial infarction, CD3, anti-CD, antibody treatment, cardioprotection, AMI, apoptosis, angiogenesis, miRNA

## Abstract

Introduction: Antibody treatment with anti-thymocyte globulin (ATG) has been shown to be cardioprotective. We aimed to evaluate which single anti-T-cell epitope antibody alters chemokine expression at a level similar to ATG and identified CD3, which is a T-cell co-receptor mediating T-cell activation. Based on these results, the effects of anti-CD3 antibody treatment on angiogenesis and cardioprotection were tested in vitro and in vivo. Methods: Concentrations of IL-8 and MCP-1 in supernatants of human peripheral blood mononuclear cell (PBMC) cultures following distinct antibody treatments were evaluated by Enzyme-linked Immunosorbent Assay (ELISA). In vivo, anti-CD3 antibodies or vehicle were injected intravenously in rats subjected to acute myocardial infarction (AMI). Chemotaxis and angiogenesis were evaluated using tube and migration assays. Intracellular pathways were assessed using Western blot. Extracellular vesicles (EVs) were quantitatively evaluated using fluorescence-activated cell scanning, exoELISA, and nanoparticle tracking analysis. Also, microRNA profiles were determined by next-generation sequencing. Results: Only PBMC stimulation with anti-CD3 antibody led to IL-8 and MCP-1 changes in secretion, similar to ATG. In a rat model of AMI, systemic treatment with an anti-CD3 antibody markedly reduced infarct scar size (27.8% (Inter-quartile range; IQR 16.2–34.9) vs. 12.6% (IQR 8.3–27.2); *p* < 0.01). The secretomes of anti-CD3 treated PBMC neither induced cardioprotective pathways in cardiomyocytes nor pro-angiogenic mechanisms in human umbilical vein endothelial cell (HUVECs) in vitro. While EVs quantities remained unchanged, PBMC incubation with an anti-CD3 antibody led to alterations in EVs miRNA expression. Conclusion: Treatment with an anti-CD3 antibody led to decreased scar size in a rat model of AMI. Whereas cardioprotective and pro-angiogenetic pathways were unaltered by anti-CD3 treatment, qualitative changes in the EVs miRNA expression could be observed, which might be causal for the observed cardioprotective phenotype. We provide evidence that EVs are a potential cardioprotective treatment target. Our findings will also provide the basis for a more detailed analysis of putatively relevant miRNA candidates.

## 1. Introduction

In spite of advances in reperfusion therapy, ischemic cardiomyopathy after acute myocardial infarction (AMI) still causes high morbidity [1]. Medical management of heart failure due to reduced ejection fraction has dramatically improved outcomes, but the great hope put in stem cell therapy to improve cardiac output has been dampened by inconsistent results from randomized clinical trials [2,3,4,5,6].

Anti-thymocyte globulin (ATG) has been shown to have cardioprotective, pro-angiogenic, and chemotactic effects in a rat model of acute myocardial infarction (AMI) [7]. ATG was formerly used in transplantation medicine and is known to induce apoptosis in T-cells, depletion of T-cell depletion and T-cell activation, depletion of antigen-presenting cells, and to induce regulatory T-cells and natural killer T cells [8,9]. ATG administration induces “paracrine effects” in cardiomyocytes in AMI [7]. The “paracrine paradigm” was developed after inconsistent results in large “stem cell therapy” trials [10,11]. Recently, Vagnozzi et al. underscored the merit of this concept and showed that cardiac “stem cell therapy” induces a wound healing and inflammatory cascade [12]. The results of our research and research by others have shown that not only secretomes of bone marrow-derived stem cells (BMCs) but also of peripheral blood mononuclear cells (PBMC) exert angiogenic and cardioprotective effects similar to effects seen in “stem cell therapy” [7,13,14,15]. 

Several potential mechanisms have been discussed to mediate the cardioprotective, angiogenic, and cardiac remodeling effects observed in therapeutic approaches using “stem cell” secretomes. Differential cell culture conditions led to changes in the secretomes of both BMCs and PBMCs, e.g., in the concentration of interleukin-8 (IL-8) which was shown to induce angiogenesis, to be cardioprotective and to inhibit hypoxia-induced apoptosis in HUVECs [16]. Cardioprotective signaling pathways induced by IL-8 were mediated via Akt1 and ERK1/2 [17,18]. Furthermore, IL-8 is known to promote angiogenesis via endothelial progenitor cell homing [19]. Other chemokines such as Interleukin-6 (IL-6) and tumor necrosis factor-alpha (TNFα) were found to induce angiogenesis as well, besides their pro-inflammatory effects [20,21]. 

Besides chemokines and cytokines, extracellular vesicles (EVs) have been identified as mediators of cardioprotective and angiogenic effects. Newly described key players in exosome-mediated effects are non-coding RNAs such as microRNA (miRNA), some of which exert profound cardioprotective effects [22,23].

Thus, paracrine effects induced by T-cell stimulation may represent a promising concept. Nevertheless, the administration of ATG in AMI is precluded in clinical practice due to known adverse reactions such as anaphylaxis, thrombocytopenia, hypotension and especially cytokine release syndrome. On the other hand, in the acute setting of AMI, a ready-to-use antibody might still be preferable over-complicated stem cell therapies necessitating individual stem cell purification and preparation. 

An anti-CD3 antibody has emerged as an alternative candidate, among other antibodies. Activation of the CD3 T-cell co-receptor induces activation, proliferation, and alters cytokine production of T-cells [24,25]. From a clinical standpoint, an anti-CD3 antibody was recently successfully evaluated in patients at risk for type-1 diabetes, evidencing clinical safety [26].

Based on these considerations and previous work, we, therefore, aimed to evaluate (1) which single anti-T-cell-epitope antibody alters chemokine expression at a similar or superior level compared to ATG, (2) if treatment using this antibody is cardioprotective in an animal model of AMI, (3) if potential effects are mediated by angiogenesis, chemotaxis and cardio-protection and (4) if antibody treatment leads to quantitative and/or qualitative alterations in the exosome secretion profile of PBMCs.

## 2. Materials and Methods

### 2.1. Cell Culture of Human PBMC

All experimental procedures involving human cells were approved by the local ethics committee (415-E/1895/4-2015) and were conducted in compliance with the Declaration of Helsinki Principles. Human peripheral blood mononuclear cells (PBMC) were obtained from young healthy volunteers after informed written consent. Inclusion criteria were: body mass index 18–40 kg/m^2^, no autoimmune disease, no intake of anti-inflammatory drugs during the last month, no sign of acute infection during the previous month, and no chronic inflammatory disease. PBMC were separated using the Ficoll-Paque technique (Ficoll^®^ Paque Plus, Sigma Aldrich, Vienna, Austria) density gradient centrifugation as described previously [16]. Cells were resuspended in X-VIVO™ 10 Chemically Defined, Serum-free Hematopoietic Cell Medium (Lonza, Switzerland) and cultured in a humidified atmosphere for 24 h at a density of 1.0 × 10^6^ cells/mL, n = 10). For cell culture experiments, ATG (Thymoglobulin, Genzyme, Germany) was added at various concentrations from 5 to 250 µg/mL. Additional dose titration experiments were conducted with increasing concentrations of anti-CD3 antibodies (5 µg, 10 µg and 25 µg).

In experiments to identify the specific T cell epitope that, once co-incubated with 20 µg/mL antibodies, induced the most pronounced chemokine secretion, the following T-cell epitopes were screened: CD4, CD8, CD11a, CD3, CD28, CD2, and HLA-DR. ATG (20 µg/mL) served as a positive control and untreated cells as well as the use of isotype antibodies (IgG1 and IgG2a) served as negative controls. All antibodies were obtained from BD Biosciences, Heidelberg, Germany.

### 2.2. Membrane Proteome Analysis

The obtained supernatants were screened for cytokines and angiogenic factors using commercially available antibody array systems (Human XL Cytokine Array Kit Proteome Profiler Array, Catalog Number ARY022B, R&D Systems, Minneapolis, USA) according to the manufacturer’s instructions. In short, capture and control antibodies have been pre-spotted in duplicate on nitrocellulose membranes. The cell culture supernatants of controls (unstimulated PBMCs, pooled supernatants) or ATG stimulated cells were added to and incubated overnight with the Proteome Profiler Array membrane on a rocking platform shaker. After the incubation period, the membrane was washed to remove unbound material followed by incubation with a cocktail of biotinylated detection antibodies. Streptavidin-HRP and chemiluminescent detection reagents were then applied, producing a signal at each capture spot corresponding to the amount of protein-bound. For analysis, membranes were transferred to an autoradiography film cassette and a Western blot imaging device ChemiDoc XRS imager (Bio-Rad, Hercules, CA, USA) for a total of nine minutes.

### 2.3. ELISA Analysis

Levels of selected cytokines secreted into the supernatant by PBMCs co-incubated with ATG or other antibodies were measured by using commercially available enzyme-linked immunosorbent assays (ELISA; Duoset, R&D Systems, Minneapolis, MN, USA) kits for the quantification of IL-8, MCP-1 and Angiogenin according to the manufacturer’s protocol. ELISA assays were performed in accordance with instructions supplied by the manufacturer. In short, cell culture supernatant samples and standard proteins were added to the multi-well plate coated with the respective capture antibody and incubated for two hours. Plates were then washed using washing buffer (Tween 20, and phosphate-buffered saline solution, Sigma Aldrich, St.Louis, MO, USA). In the next step, a biotin-labeled antibody was added to each well and incubated for another two hours. ELISA plates were washed again, and a streptavidin-horseradish-peroxidase solution was added. After adding tetramethylbenzidine (TMB; Sigma Aldrich, USA), a color reaction was achieved. Optical density was measured at 450 nm on an ELISA plate-reader (iMark Microplate Absorbance Reader, Bio-Rad Laboratories, Austria).

### 2.4. Human Cardiomyocyte Culture and Immunoblot Analysis

Human cardiomyocytes (HCMs; Promo-Cell, Heidelberg, Germany) were cultivated in myocyte growth medium supplemented with supplement mix (PromoCell), 1% L-glutamine 1% PenStrep in T75 flasks. For Western blot analysis, cells were seeded at a concentration of 2 × 105 cells in a 6-well plate.

In the case cells were not confluent, 3 mL of fresh cultivation medium was added. Twenty-four hours later, the cultivation medium was replaced for basal medium for starvation medium (supplemented only with 1% PenStrep and 1% L-glutamine) for 3 h. Then HCMs were treated for 60 min with one of the three different conditioned media: PBMC ± 250 µL/mL ATG (pool supernatant), PBMC ± 10 µg/mL anti-CD3 (pool supernatant), PBMC ± 10 µg/mL Isotype (pool supernatant) or with Myocyte growth media.

Immunodetection was performed for the protein expression of the total and the phosphorylated form of Akt and ERK 1/2 in HCMs by Western immunoblotting. 200 µL of RIPA buffer supplemented with Phosphatase inhibitor were added to each well for the total protein isolation and quantified the content of the extracts using Bradford assays. Proteins were then separated on Bolt 10% Bis-Tris Plus Gel (Novex, Life Technologies, Carlsbad, CA, USA) and transferred onto nitrocellulose membranes. Membranes were blocked for one h at room temperature (5% milk powder in TBS ± 0.1% Tween). The following primary antibody (all from Cell Signaling Technology, Danvers, MA, USA) were used for incubation overnight at 4 °C: Phospho-Akt (Ser473, 1:1000), Akt (pan) (C67E7, rabbit mAb, 1:1000), Phospho-p44/42 MAPK (Erk1/2) (Thr202/Tyr204), rabbit mAb, 1:2000, p44/42 MAPK (Erk1/2) (137F5), rabbit mAb, 1:1000.

This incubation was followed by the addition of the horseradish peroxidase (HRP) Envision± labeled anti-mouse or anti-rabbit antibody (Dako, Santa Clara, CA, USA) diluted 1:500 in TBS-T ± 5% Bovine Serum Albumin (BSA Sigma Aldrich, St.Louis, MO, USA). Band intensity from the membranes was analyzed using the GE Healthcare Amersham™ ECL™ Prime Western Blotting Detection Reagent 1:1 (GE Healthcare Life Sciences, Chicago, Illinois, USA) and the ChemiDoc XRS imager (Bio-Rad, Hercules, CA, USA) and measurements were repeated three times. Confirmation of equal loading was done by the expression of α-actinin (1∶1000, Santa Cruz Biotechnology, Dallas, TX, USA).

### 2.5. Human Umbilical Cord Endothelial Cell Assays

#### 2.5.1. Tube Formation Assay

Twenty-four-well plates were coated with 300 µL Matrigel (Thermo Fisher Scientific, Waltham, MA, USA) and incubated for one hour at 37 °C. Following this, 1 × 10^5^ human umbilical vein endothelial cells (HUVEC, Thermo Fisher Scientific) were treated with one of the following secretomes or media: PBMC plus 10 µg/mL CD3 (pooled supernatant), PBMC plus 10 µg/mL Isotype (pooled supernatant). Medium 200 (Thermo Fisher Scientific) served as negative controls. The tube formation was visualized and photographed using an inverted photomicroscope.

#### 2.5.2. Scratch Migration Assay

For cell motility assay, the cultured human umbilical vein endothelial cells (HUVEC, Thermo Fisher Scientific, Germany) were seeded at 4 × 104 cells/well into 2-well silicone inserts with a defined cell-free gap, suitable for wound healing/migration assays (IBIDI, Gräfelfind, Germany). After 24 h of incubation at 37 °C with 5% CO2, the silicone insert was gently removed using a tweezers and the cells were washed with Medium 200 supplemented with LVES (Thermo Fisher Scientific, Waltham, MA, USA) to remove the un-adherent cells.

Subsequently, the cells were treated with different supernatants and media to evaluate their impact on gap closure. The growth process over the gap was monitored at 0, 3, 6, 9, 12 and 24 h by measuring cell confluence in a pre-defined, constant area including the gap using a Spark^®^ 10M multimode microplate reader (Tecan, Anif, Austria). The gap width was quantified using ImageJ (v1.52i) software (National Institutes of Health, Bethesda, MD, USA).

### 2.6. Experimental Rat Model of Acute Myocardial Infarction

Animal experiments were approved by the Committee for Animal Research, Medical University of Vienna 66.009/0122-WF/V/3b/2017. All experiments were performed in accordance with the Guide for the Care and Use of Laboratory Animals by the National Institutes of Health (NIH Publication No. 85-23, revised 1996). Myocardial infarction was induced by ligation of the left coronary artery (LCA) as described previously [27,28]. Briefly, male Sprague-Dawley rats were anesthetized by intraperitoneal injection of mixture of Xylazine (4 mg/kg; Bayer, Germany) and Ketamine (100 mg/kg; Dr. E. Gräub AG, Switzerland), intubated (14-gauge tube) and ventilated (9 mL/kg body weight, 75–85 stroke/min). Rats allocated to (1) MI (n = 12 and (2) MI plus anti-CD3 group (n = 13) and were subjected to permanent left coronary artery (LCA) ligation using 6-0 prolene. Rectal temperature was measured and maintained at 37.5–38.5 °C by a heated operating table. Myocardial ischemia was associated with cyanosis of the myocardial area at risk and ST-elevation on the ECG signal. Analgesia was initiated by intraperitoneal injection of Piritramide (0.1 mL/kg body weight) preoperatively and Piritramide in drinking water was applied as a postoperative analgesic regimen (2 ampules of Piritramide with 30 mL of Glucose 5% in 250 mL water). Fifty micrograms of anti-CD3 antibodies suspended in 0.3 mL 0.9% saline solution was injected into the femoral vein as a single dose. Injection of saline solution (vehicle) alone and sham operation served as controls.

### 2.7. Histology

Rats were sacrificed six weeks after experimental infarction. Hearts were explanted and then sliced at three layers at the level of the largest extension of infarcted area (n = 12 for 6 weeks analyses in the control group, n = 13 in anti-CD3 antibody treatment). Slices were fixed in 10% neutral buffered formalin and embedded in paraffin. The tissue samples were stained with hematoxylin-eosin (H&E) and van Gieson (VG). Tissue samples were evaluated on an inverted microscope at 200× or 400× magnification. Image J planimetry software (Rasband, W.S., Image J, U.S. National Institutes of Health, Bethesda, USA) was utilized to determine the area of necrosis after three days and the size of myocardial infarction after 6 weeks. Six weeks after induction of experimental AMI, a planimetric evaluation was carried out on tissue specimens stained with VG for a better comparison of vital myocardium and fibrotic areas. Infarction size was expressed as a percentage of the total left ventricular area as previously described [29].

### 2.8. Flow Cytometry (FACS) Analysis

Extracellular vesicles (EVs) in the collected cell culture supernatant samples obtained after stimulation with either 20 µg anti-CD3, 250 µg ATG antibodies, and untreated controls were stained with antibodies against CD9, CD63, CD81 and HSP70 for analysis in flow cytometry.

For this, 75 µL microliters of supernatant were incubated for 15 min with labeled antibodies: 10 µL of CD81 FITC (BD Biosciences, Heidelberg, Germany), 1 µL of HSP70 PE (Abcam, Cambridge, United Kingdom), 5 µL of CD63 PE-Cy7 (BD Biosciences), and 5 µL of CD9 APC (BD Biosciences), or matching isotype controls (FITC, PE, and APC obtained from Life Technologies, Darmstadt, Germany; PE-Cy7 obtained from BD Biosciences). In the next step, samples were then diluted with 400 µL phosphate-buffered saline. To determine the number of the acquired events, size calibration beads were added to the samples (flow cytometry size calibration kit 1–15 µm from Life Technologies). The samples were analyzed in a flow cytometer (FACS Calibur, BD Biosciences). MP was defined as particles smaller than 1.5 µm and appropriately gated. MPs positive for the respective antibodies were expressed as the percentage of events in the relevant gate. The obtained data were processed using CellQuest Pro software (BD Biosciences).

### 2.9. ExoELISA CD81

Supernatants of ATG and anti-CD3 treated PBMCs were analyzed for concentrations of CD81 positive EVs using the commercially available ExoELISA CD81 kit (System Biosciences, Palo Alto, CA, USA). In brief, EVs were captured on a protein binding microtiter plate precoated with an anti-CD81 primary antibody recognizing the tetraspanin protein on the exosomal surface. A Horseradish Peroxidase enzyme-linked secondary antibody was used for signal amplification, before adding a colorimetric TMB substrate solution. Color intensities were quantitated at 450 nm in an ELISA plate-reader (iMark Microplate Absorbance Reader, Bio-Rad Laboratories, Austria).

### 2.10. Isolation of Total RNA and Preparation of Small RNA Libraries

EVs were purified by centrifugation at 100,000× *g* for 1.5 h at 4 °C. The pellet was lysed with 1000 µL Qiazol, supplemented with 1 µL of a synthetic RNA mixture containing three different synthetic control RNAs (UniSp2, two fmol/mL; UniSp4, 0.02 fmol/mL; UniSp5, 0.0002 fmol/mL; Qiagen, Hilden, Germany). RNA extraction was performed using 200 µL chloroform, and phase separation was achieved by centrifugation for 15 min at 12,000× *g* at 4 °C. RNA was extracted from the upper aqueous phase and purified on a QIAcube liquid handling robot using the miRNeasy Mini kit (Qiagen) with the following modifications: glycogen (Ambion, Austin, TX, USA) was added to the aqueous phase to a final concentration of 50 mg/mL and precipitated with 750 µL 100% ethanol. Columns were washed twice with RPE buffer and RNA was eluted in a single round in 30 µL nuclease-free water and stored at –80 °C. 

Small RNA Libraries were prepared from 2 µL RNA, using the CleanTag Small RNA Library Prep Kit (TriLink Biotechnologies, San Diego, CA, USA) according to the manufacturer’s protocol. cDNA libraries were amplified in 21 PCR cycles. Equimolar amounts were pooled and libraries underwent 50 cycles of single-end sequencing on a HighSeq 2500 (Illumina, San Diego, USA).

### 2.11. Nanoparticle Tracking Analysis (NTA)

Nanoparticle tracking analysis (NTA) was performed on purified PBMC-derived EV samples to determine particle size and concentration using the ZetaView^®^ PMX-120 instrument according to the instructions of the manufacturer.

### 2.12. Next-Generation Sequencing Data Analysis

Raw reads were adapter trimmed using cutadapt, and quality checked using fastqc (v0.10.1). Reads with sufficient length (> 18 nt) and quality (phred > 30) were sequentially mapped against all human mature microRNA sequences (miRBase v20) and the human genome (GRCh37). MicroRNA reads were normalized to the total library size to yield reads per million (RPM) for visual representation. Raw read counts were subsequently used in EdgeR to identify microRNAs differentially expressed in extracellular vesicles derived from anti-CD3 versus Isotype control-treated PMBCs, applying modified paired t-tests [30]. Principal component analysis and hierarchical clustering were performed using miRNA RPM values and clustvis (https://biit.cs.ut.ee/clustvis/).

### 2.13. Statistical Analysis 

Statistical analysis was performed using GraphPad Prism software (GraphPad Prism version 8.00 for Windows, GraphPad Software, San Diego California USA, www.graphpad.com”). All data are given as mean ± SEM. The repeated ANOVA and one-way analysis of variance or Kruksal-Wallis test were utilized accordingly to calculate significances between the groups. The Dunnett’s and the Dunn’s multiple comparison post-tests were utilized, and the Benjamini-Hochbergs method for false-discovery rate (FDR) to adjust for multiple testing was applied as appropriately. Generally, *p*-values < 0.05 were considered statistically significant (*p*-values were expressed as follows: * *p* < 0.05, ** *p* < 0.01, *** *p* < 0.001).

## 3. Results

### 3.1. Cell Culture Supernatants Obtained from PBMCs Co-Incubated with ATG and Anti-CD3 Antibody Show High Concentrations of Chemokines

**Figure 1:**
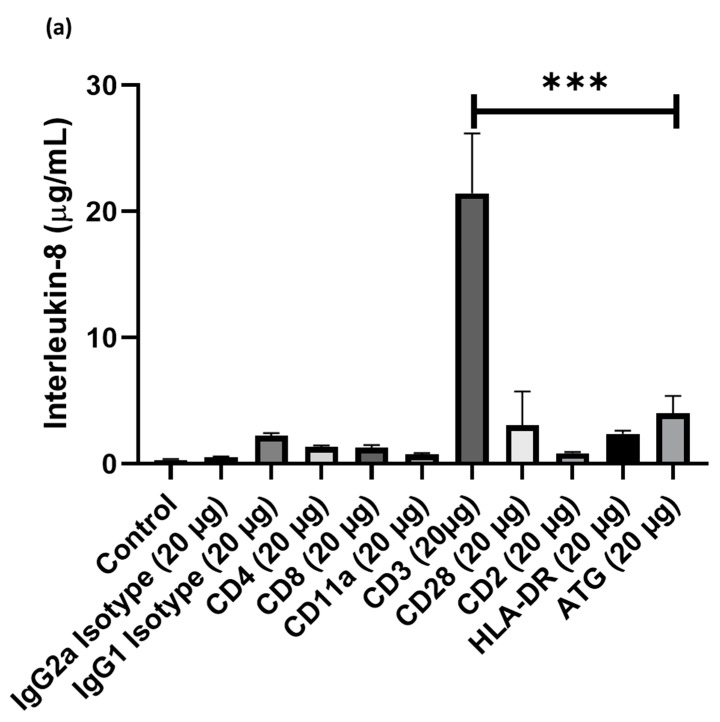

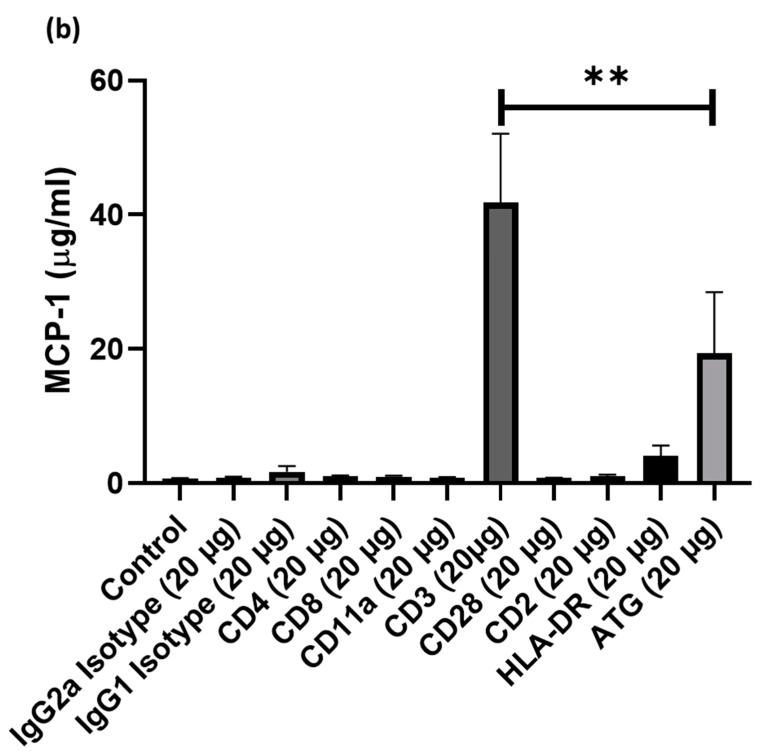
Cell cultures of human peripheral blood mononuclear cell (PBMC)were supplemented with different doses of Anti-thymocyte globulin (ATG) or various anti-T-cell antibodies, such as treatment with anti- (1) IgG2a, (2) IgG1, (3) CD4, (4) CD8, (5) CD11a, (6) CD3, (7) CD28, (8) CD2, (9) HLA-DR. After 24 h incubation, supernatants were collected and the concentration of IL-8 and MCP-1 was assessed by enzyme-linked immunosorbent assays (ELISA). Only the addition of anti-CD3 antibodies led to a pronounced (**a**) IL-8 and (**b**) MCP-1 expression compared with other epitopes in PBMCs. (*p*-values were expressed as follows: ** *p* < 0.01, *** *p* < 0.001).

Cell cultures of human PBMCs were supplemented with different doses of ATG or various anti-T-cell antibodies, such as treatment with anti- (1) IgG2a, (2) IgG1, (3) CD4, (4) CD8, (5) CD11a, (6) CD3, (7) CD28, (8) CD2, (9) HLA-DR. After 24 h incubation, supernatants were collected and the concentration of IL-8 and MCP-1 was assessed by ELISA. Compared to ATG (4005 ± 1378 pg/mL), only the addition of anti-CD3 antibodies led to significantly increased IL-8 expression (21438 ± 14990 pg/mL; *p* < 0.001 vs. ATG; Figure 1a), whereas the addition of anti-IgG2a (531 ± 193 pg/mL; p = n.s.), anti-IgG1 (22231 ± 666 pg/mL p = n.s.), anti-CD4 (1327 ± 140 pg/mL; p = n.s.), anti-CD8 (1298 ± 181 pg/mL; p = n.s.), anti-CD11a (751 ± 104 pg/mL; p = n.s.), anti-CD28 (3062 ± 2673 pg/mL; p = n.s.), anti-HLA-DR (2375 ± 263 pg/mL; p = n.s.) did not lead to higher IL-8 concentrations in the PBMC supernatant. Similarly, the incubation with anti-CD3 (41835 ± 10241 pg/mL; *p* < 0.01) led to increased MCP-1 concentrations in the PBMC supernatant, which were markedly higher compared to ATG (19399 ± 9078 pg/mL). In contrast, all other antibodies did not lead to appreciable increases in MCP-1 levels. For proof of concept of previous work showing an increased secretion of chemokines after ATG-stimulation in PBMCs, PBMCs were co-incubated with ATG versus control and concentrations of chemokines in supernatants were quantified using an array. Concentrations of several chemokines, including IL-8, showed marked alterations upon treatment of PBMCs with ATG (Supplemental Figure S1).

### 3.2. Effects of Systemic Intravenous Anti-CD3 Treatment on Myocardial Scar Size

**Figure 2:**
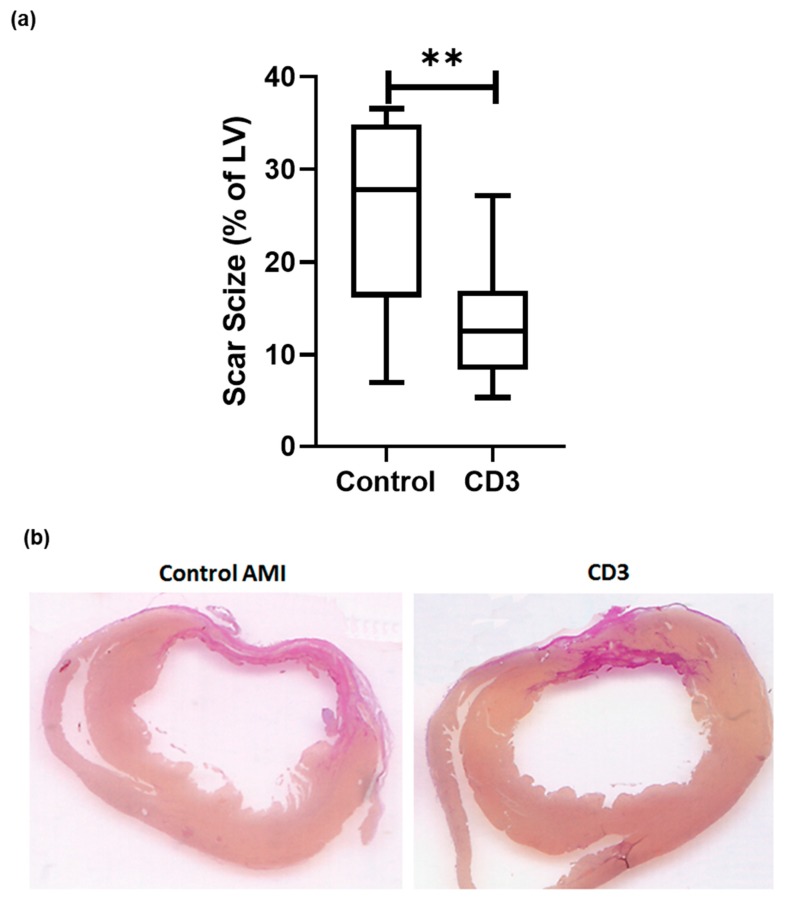
Rats were subjected to permanent left coronary artery (LCA) ligation. Rats were sacrificed 6 weeks after experimental infarction. Hearts were explanted and then sliced at three layers at the level of the largest extension of infarcted area and infarct sizes compared after staining with hematoxylin-eosin (H&E) and van Gieson (VG). (**a**) In animals treated with anti-CD-3 antibody (n = 13) left ventricular scar size was significantly reduced [12.6% (IQR 8.3–27.2) vs. 27.8% (IQR 16.2–34.9) *p* < 0.01] compared to vehicle treatment (n = 12). Representative (**b**) pictures are shown. (*p*-values were expressed as follows: ** *p* < 0.01).

As described above, experimental myocardial infarction was evaluated in a rat model, subjected to left coronary artery ligation. Six weeks after the induction of acute myocardial infarction and subsequent anti-CD3 treatment, hearts were explanted, and myocardial scar size was determined. The hearts obtained from rats treated with anti-CD3 antibodies evidenced smaller scar formation. Accordingly, a planimetric analysis showed a scar size of 27.8% (IQR 16.2-34.9) of the left ventricle in control animals, while this was significantly reduced to 12.6% in the specific treatment group (IQR 8.3-27.2, *p* < 0.01, n = 12–13 per group, Figure 2a, representative pictures in Figure 2b).

### 3.3. Induction of Chemotaxis and Angiogenesis by the Secretomes of Anti-CD3 Treated PBMC 

**Figure 3:**
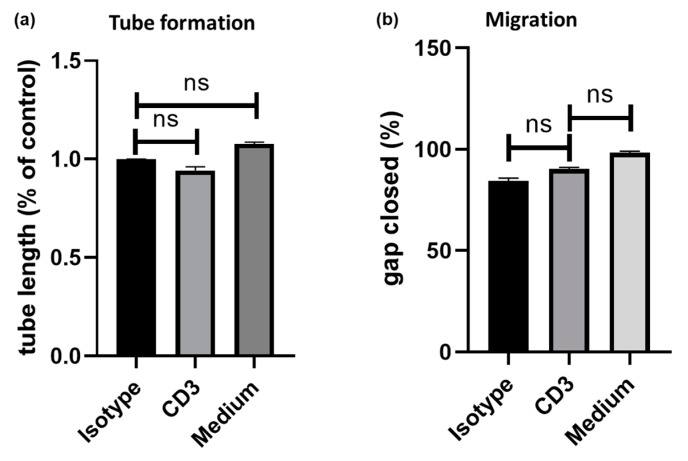
human umbilical vein endothelial cell (HUVECs) were exposed to anti-CD3 treated PBMCs. Cell migration and angiogenesis were assessed by (**a**) tube assay and (**b**) tube formation, respectively. Compared to isotype control (100%), exposure to supernatant of anti-CD3 treated PBMC did not induce (**a**) endothelial cell tube formation (94 ± 2%; p = n.s. versus isotype control) (**b**) nor chemotaxis (90 ± 1%; p = n.s.) in HUVECs.

The effects of exposure of HUVECs to secretomes of PBMC after anti-CD3 treatment were evaluated in both a scratch assay for studying cell migration and in a tube assay for the assessment of promotion or inhibition of tube formation as a surrogate parameter of angiogenesis. Compared to isotype control (100%), exposure to PBMC supernatant after anti-CD3 antibody (94 ± 2%; p = n.s. versus isotype control), medium alone (108 ± 1%; p = n.s. versus isotype control) and medium ± RPMI (98 ± 2%; p = n.s. versus isotype control) did not induce tube formation (Figure 3a). Similarly, gap closure in HUVECs was not increased by coincubation with PBMC supernatant after anti-CD3 antibody treatment (90 ± 1%; p = n.s.) compared to an isotype control (84 ± 2%) and medium alone (98 ± 1%; p = n.s.; Figure 3b). Furthermore, exposure to PBMC supernatant after anti-CD3 antibody treatment did not increase angiogenin secretion in HUVECs compared to an isotype control (800 ± 200 pg/mL versus 852 ± 105 pg/mL, p = n.s.).

### 3.4. Effects of Exposure to Anti-CD3 Treated PBMC Supernatant on Intracellular Pathways in Human Cardiomyocytes

**Figure 4:**
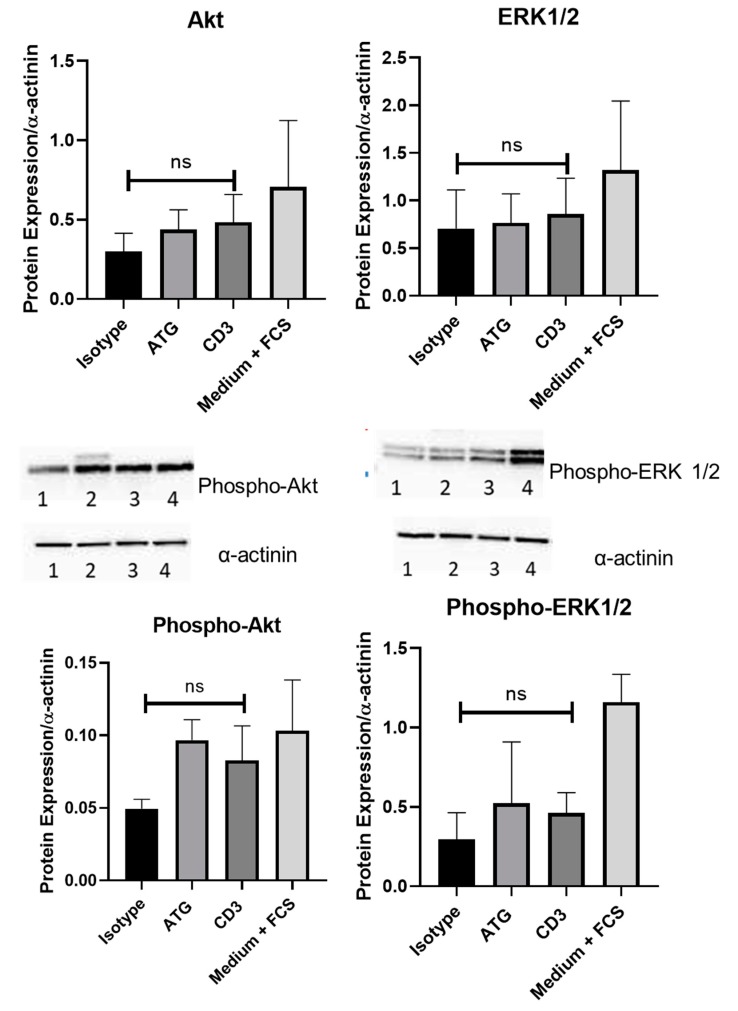
The effect of anti-CD3 treatment on intracellular pathways was investigated by Western Immunoblotting. In human cardiomyocytes, co-incubation with supernatant of PBMCs treated with anti-CD3 antibody did not alter phosphorylation of Akt (0.05 ± 0.01 versus 0.08 ± 0.02 protein expression/alpha-actinin; p = n.s.) and ERK1/2(0.30 ± 0.17 versus 0.46 ± 0.13 protein expression/alpha-actinin; p = n.s) compared to isotype control. Representative pictures of the isotype (1), ATG (2), anti-CD3 (3) and medium plus FCS (4) conditions are shown.

The effect of anti CD3 treatment on intracellular pathways was investigated by Western Immunoblotting. Treatment with PBMC supernatant after anti-CD3 antibody treatment did not alter phosphorylation of ERK1/2 (0.30 ± 0.17 versus 0.46 ± 0.13 protein expression/alpha-actinin; p = n.s) and Akt (0.05 ± 0.01 versus 0.08 ± 0.02 protein expression/alpha-actinin; p = n.s.; all *p*-values versus CD3) compared to isotype control in human cardiomyocytes, or compared to ATG (p-ERK1/2 0.52 ± 0.39 protein expression/alpha-actinin; p = n.s.; p-Akt 0.10 ± 0.01 protein expression/alpha-actinin; p = n.s.; all *p*-values versus CD3) or medium (ERK1/2 1.16 ± 0.18 protein expression/alpha-actinin; pAkt 0.10 ± 0.04; p = n.s.; all *p*-values versus CD3) treatment (Figure 4).

### 3.5. FACS Analysis of Exosomes Markers on EVs of PBMC after Incubation with Anti-CD3 Antibodies

**Figure 5:**
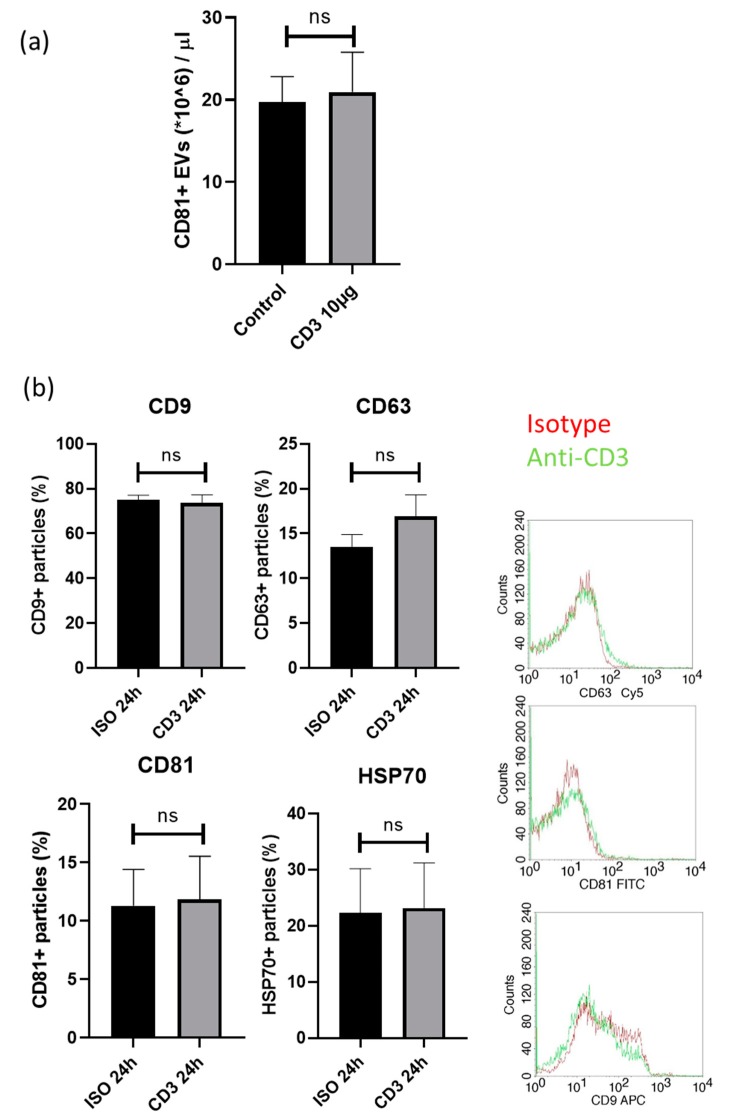
Exosome markers were assessed by (**a**) exoELISA and (**b**) FACS. The expression of the exosome marker (**a**) CD81 was not different (20.94 ± 4.84 × 10^6^ exosomes/µL versus 39.36 ± 3.14 × 10^6^ exosomes/µL; p = n.s.) compared to isotype control. The expression (**b**) of exosomes positive for CD9 (74 ± 4% versus 75 ± 2%; p = n.s), HSP70 (22 ± 8% versus 23 ± 8%; p = n.s) or CD81 (11 ± 3% versus 12 ± 4%; p = n.s.) and CD63+ exosomes (14 ± 1% versus 17 ± 2; p = n.s.) was unchanged upon anti-CD3 antibody treatment compared to isotype control.

Using exoELISA, we assessed the concentration of CD81 positive exosomes: There were no quantitative changes in the numbers of exosomes positive for the exosome marker CD81 (20.94 ± 4.84 × 10^6^ exosomes/µL versus 39.36 ± 3.14 × 10^6^ exosomes/µL; p = n.s.; Figure 5a), indicating that the quantitative EV secretion in PBMCs is not affected by anti-CD3 antibodies.

These findings were also confirmed by FACS analysis for the assessment of EV markers (HSP70, CD9, CD63 and CD81) on EVs of cultured PBMC after co-incubation with anti-CD3 antibodies (Figure 5b). The expression of exosomes positive for CD9 (74 ± 4% versus 75 ± 2%; p = n.s), HSP70 (22 ± 8% versus 23 ± 8%; p = n.s) or CD81 (11 ± 3% versus 12 ± 4%; p = n.s.) was unchanged, while a slight, but not significant increase in CD63+ exosomes (14 ± 1% versus 17 ± 2; p = n.s.) was detected upon anti-CD3 antibody treatment compared to isotype control. Concomitantly, using NTA, no differences in extracellular vesicle size and concentration were detected (data not shown).

### 3.6. miRNA Screening in EVs and Alterations in miRNA Signaling Caused by Anti-CD3 Stimulation

**Figure 6:**
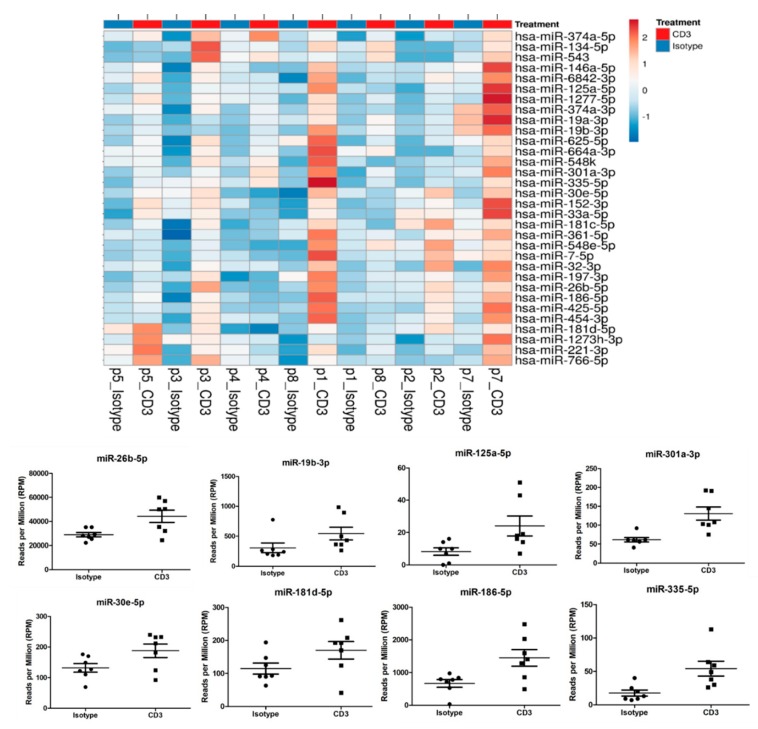
miRNA contents were analyzed by next generation sequencing. In total, 322 miRNAs were detected across all samples at a sufficient level (RPM > 5) and considered for statistical analysis. Anti-CD3 stimulation led to alterations in 32 miRNAs (10%) compared to supernatant of non-stimulated PBMC, including miRNAs miR-19a-3p and miR-19b-3p, miR-26b-5p, miR-30e-5p, miR125a-5p, miR-181d-5p, miR-186-5p, miR-301a-3p and miR-335-5p.

Despite EV numbers being unaffected by anti-CD3 antibody treatment, they may still contain different contents, including miRNAs. This finding prompted us to analyze miRNA contents by next-generation sequencing. Three hundred twenty-two miRNAs were detected across all samples at a sufficient level (RPM > 5) and considered for statistical analysis. Out of these, 32 miRNAs (10%) showed a significant change upon anti-CD3 stimulation (Table 1 and Figure 6). These include miRNAs miR-19a-3p and miR-19b-3p, miR-26b-5p, miR-30e-5p, miR125a-5p, miR-181d-5p, miR-186-5p, miR-301a-3p and miR-335-5p, which have been described as relevant in cardiovascular context (see Discussion). Thus, our findings will also provide the basis for the more detailed analysis of putatively relevant miRNA candidates.

## 4. Discussion

In this study, (1) systemic intravenous treatment with an anti-CD3 antibody markedly reduced scar formation in a rat model of AMI, (2) exposure to PBMC supernatant after anti-CD3 antibody treatment did neither induce cardioprotective pathways in human cardiomyocytes, nor pro-angiogenetic mechanisms in vitro in HUVECs and (3) PBMC incubation with an anti-CD3 antibody led to a qualitative but not quantitative change of the exosome and miRNA expression.

Ischemic cardiomyopathy due to acute myocardial infarction (AMI) constitutes a challenge to patients and health systems [1]. The initial hope that stem cell therapy has a benefit was disappointed by several large clinical trials. [2,3,4,5]. Consequently, the so-called “paracrine paradigm” was formulated stating not the stem cells themselves but their secretomes are responsible for some of the promising results in clinical trials investigating “stem cell” therapy [10,11]. Several mechanisms including infusion of apoptotic cell suspensions after irradiation, were shown to be cardioprotective and reduce infarct size, underscoring the theoretical merit of the “paracrine paradigm” [31,32,33]. The “stem cells” likely mediated the observed effects through their secretomes and not via cellular incorporation and direct tissue regeneration [34]. Therefore, the infusion of “stem cells” per se might be unnecessary [35].

Whereas treatment with ATG was shown to decrease infarct size in a preclinical model of AMI in rats, using ATG in a clinical setting in patients with AMI is prohibited due to several side-effects. We, therefore, investigated a way of inducing a similar “danger signal” in PBMC through T-cell stimulation mediated through only one epitope. Using an array, we first confirmed IL-8 as a potent surrogate parameter for ATG-induced secretory changes in PBMC. We are aware that in the stem cell trials, bone-marrow-derived stem cells (BMSC) were used as opposed to PBMC and that Wollert et al. showed distinct expression patterns of PBMC and BMSC [13,14]. However, firstly, in our previous work, PBMC and BMSC were comparable regarding IL-8 secretion. Secondly, we think that although the concept of antibody treatment for cardioprotection originates from “stem cell therapy”, it now transcends this idea. PBMC were, therefore, chosen to assess this question as PBMC are comparable to stem cells with regards to exosome secretion but easier to obtain and more cost-efficient and abundant in whole blood and therefore pass the coronaries constantly, where they might work as “bioreactors” of cardioprotective factors [14].

Compared to ATG, CD3-mediated stimulation resulted in a more profound IL-8 and MCP-1 expression pattern, an established surrogate marker of changes in exosome secretion. We, therefore, concluded that specific anti-CD3 antibody treatment could theoretically combine the efficacy of ATG treatment with better safety. Of note, while an effective reduction of left ventricular scar size in an animal AMI model compared to control could be observed, the issue of safety of anti-CD3 treatment in humans suffering from AMI is beyond the scope of this study. It is noteworthy that the safety of anti-CD3 antibody treatment has been proved in a recent trial [26].

At least partly, the beneficial effects observed in our animal model of AMI might be mediated through IL-8 and cytokines. We and others showed previously that cell-free suspension of PBMC evidencing high IL-8 induced angiogenesis and cardioprotective pathways [13]. However, in this study, PBMC supernatant after anti-CD3 antibody treatment did not induce increased migration and tube formation in HUVECs. Further, angiogenin secretion was not increased in HUVEC co-incubated with supernatant of PBMCs treated with an anti-CD3 antibody. Contrary to our expectation, exposure to PBMC supernatant after anti-CD3 antibody treatment did not significantly induce known cardioprotective signaling pathways such as phosphorylation of Akt and Erk1/2 in human cardiomyocytes. Therefore, the observed effects of anti-CD3 antibody treatment in our AMI model are likely not predominantly due to classical primarily cardioprotective mechanisms but influenced by changes in other mechanisms resulting in the observed cardioprotective phenotype. In a previous study, p53, a known mediator of cardioprotection, was downregulated in a rat model of AMI after ATG treatment [7]. However, in this study, ATG also triggered the release of cytokines as well as neoangiogenesis and chemotaxis, which we could not detect in our study evaluating specific anti-CD3 treatment. We, therefore, think that ATG treatment does not only differ with regards to the applicability to anti-CD3 treatment, but also regarding the mechanistic pathways by which the observed cardioprotective phenotypes were mediated. Therefore, furthermore, both quantitative and qualitative changes in exosomes, which are known to have cardioprotective capabilities, were assessed in this study [23]. Indeed, pronounced effects on several distinct miRNAs were observed.

Several miRNA were significantly upregulated in the anti-CD3 treated PBMC supernatant. The assessment of their specific roles on the cardioprotective effect observed in this study are beyond the scope of this manuscript. The effects of miRNA in heart disease and cardio-protection are reviewed elsewhere [35,36,37,38].

Different miRNAs could contribute to this effect. miR-19 was shown to induce tissue factor in breast cancer cells [39]. Recently, Li et al. described the miR-19 family as a potential target in cardiovascular medicine [40]. miR-19 and miR-186 were evaluated in the context of coronary heart disease and the diagnosis of acute myocardial infarction [41]. miR-134 was shown to be a potential biomarker for both AMI and adverse outcome including the development of heart failure after AMI [42]. miR-26 plasma concentration was shown to be associated with AMI [43]. miR-374 was shown to be pro-hypertrophic and to be involved in VEGF-receptor-1 signaling [44]. The miR-26 family plays an essential role in angiogenesis, epithelial cell growth and tissue repair [45]. miR-30 was shown to be cardioprotective in heart failure inhibiting cardiomyocyte autophagy [46]. The miR-30 family is abundant in the heart and to participate in ventricular remodeling through autophagy, inflammatory pathways and apoptotic mechanisms [47]. miR-146a is known to be cardioprotective and to reduce infarct size in in vivo animal models of AMI [48]. miR-186 was shown to play a crucial role in vascular endothelial cell angiogenesis [49]. miR-221 was shown to inhibit autophagy after hypoxia and was postulated to be a possible target in treating ischemia-reperfusion injury [50].

The physiologic and pathophysiologic changes induced by distinct miRNA expression patterns and the clinical relevance of these miRNA alterations are beyond the scope of this manuscript. However, we speculate that the observed cardioprotective effect of anti-CD3 treatment in vivo could potentially result from alterations in miRNA expression and concentration. We provide new data for future studies evaluating miRNA alterations in the setting of cardioprotective treatment.

### Limitations

Several limitations need to be addressed. Firstly, we lack functional in vivo data such as cardiac magnetic resonance imaging and pressure measurements. Secondly, we do not have a proof for actual T-cell stimulation by the anti-CD3 in vivo treatment, though there seems to be cross-species reactivity when administrating human ATG antibodies in rats as an increase of the IL-8 analog CXCL1 was found in rats [1]. Of note, human and rat anti-CD3 antibodies were used in vitro and in vivo respectively in this study. Thirdly, the data about the physiological relevance of the qualitative change of the exosome expression pattern remains speculative and therefore thesis-generating. Fourthly, as discussed above, although Akt and Erk represent critical pathways, other cardioprotective extracellular and intracellular pathways that were not specifically evaluated in this study could also contribute to the observed phenotype. Penultimately, the in-vivo AMI model evaluating the effects of anti-CD3 antibody lacks a true randomization as well as a baseline measure of initial myocardial injury size in the anti-CD3 versus control group. Of note, however, the analysis of the infarct sizes was blinded. Finally, this study lacks data on the interplay between T-cells and other cells of the immune system such as macrophages which were reported to play a role in exogenous cell therapy but also remodeling after AMI [51,52]. To conclude, we consider this manuscript to be hypothesis-generating: Treatment with an anti-CD3 antibody led to decreased scar size in a rat model of AMI, however, not due to (1) induction of angiogenesis, (2) classical cardioprotective pathways and (3) quantitative changes in exosomes. We, therefore, conclude that subtle qualitative changes in the miRNA expression could contribute to this exploratory finding. Further studies are warranted to evaluate the thesis-generating results reported in this manuscript.

## 5. Conclusions

Treatment with an anti-CD3 antibody led to decreased scar size in a rat model of AMI. Whereas cardioprotective and pro-angiogenetic pathways were unaltered by anti-CD3 treatment, qualitative changes in the EVs miRNA expression could be observed, which might be causal for the observed cardioprotective phenotype. We provide evidence that EVs are a potential cardioprotective treatment target. Our findings will also provide the basis for a more detailed analysis of putatively relevant miRNA candidates. 

## Figures and Tables

**Table 1:** In total, 32 of 322 miRNAs showed a significant change upon anti-CD3 stimulation in next-generation sequencing. Raw read counts were used in EdgeR to identify microRNAs differentially expressed in extracellular vesicles derived from PBMCs treated with anti-CD3 versus Isotype control.

miRNA	logFC	*p*-value
hsa-miR-19a-3p	1.01	0.001
hsa-miR-374a-5p	1.46	0.003
hsa-let-7a-3p	0.50	0.004
hsa-miR-6842-3p	1.12	0.005
hsa-miR-301a-3p	1.09	0.007
hsa-miR-664a-3p	1.12	0.011
hsa-miR-181d-5p	0.57	0.011
hsa-miR-30e-5p	0.51	0.011
hsa-miR-151a-3p	0.48	0.012
hsa-miR-19b-3p	0.83	0.013
hsa-miR-1277-5p	2.23	0.015
hsa-miR-374a-3p	0.60	0.016
hsa-miR-548e-5p	1.56	0.016
hsa-miR-32-3p	1.44	0.017
hsa-miR-186-5p	1.12	0.019
hsa-miR-221-3p	0.68	0.019
hsa-miR-548k	1.71	0.022
hsa-miR-335-5p	1.62	0.026
hsa-miR-134-5p	1.27	0.027
hsa-miR-26b-5p	0.61	0.027
hsa-miR-1273h-3p	0.79	0.029
hsa-miR-425-5p	0.91	0.030
hsa-miR-766-5p	1.00	0.032
hsa-miR-125a-5p	1.53	0.037
hsa-miR-152-3p	0.87	0.037
hsa-miR-361-5p	0.70	0.037
hsa-miR-33a-5p	1.09	0.040
hsa-miR-181c-5p	0.67	0.043
hsa-miR-197-3p	0.61	0.043
hsa-miR-7-5p	0.72	0.043
hsa-miR-543	1.37	0.044
hsa-miR-625-5p	0.79	0.044

## Supplementary Materials

The following are available online at https://www.mdpi.com/2073-4409/9/2/295/s1, Figure S1: Concentrations of several chemokines, including IL-8, showed marked alterations upon treatment of PBMCs with ATG.
