# Supplementary material for: Anti-CD3 Antibody Treatment Reduces Scar Formation in a Rat Model of Myocardial Infarction
Source: Cells. 2020 Jan 25;9(2):295. doi: 10.3390/cells9020295 (PMC7072364; doi:10.3390/cells9020295)
Supplement: Supplementary file 1 [file cells-09-00295-s001.pdf]

**Supplementary Figure**

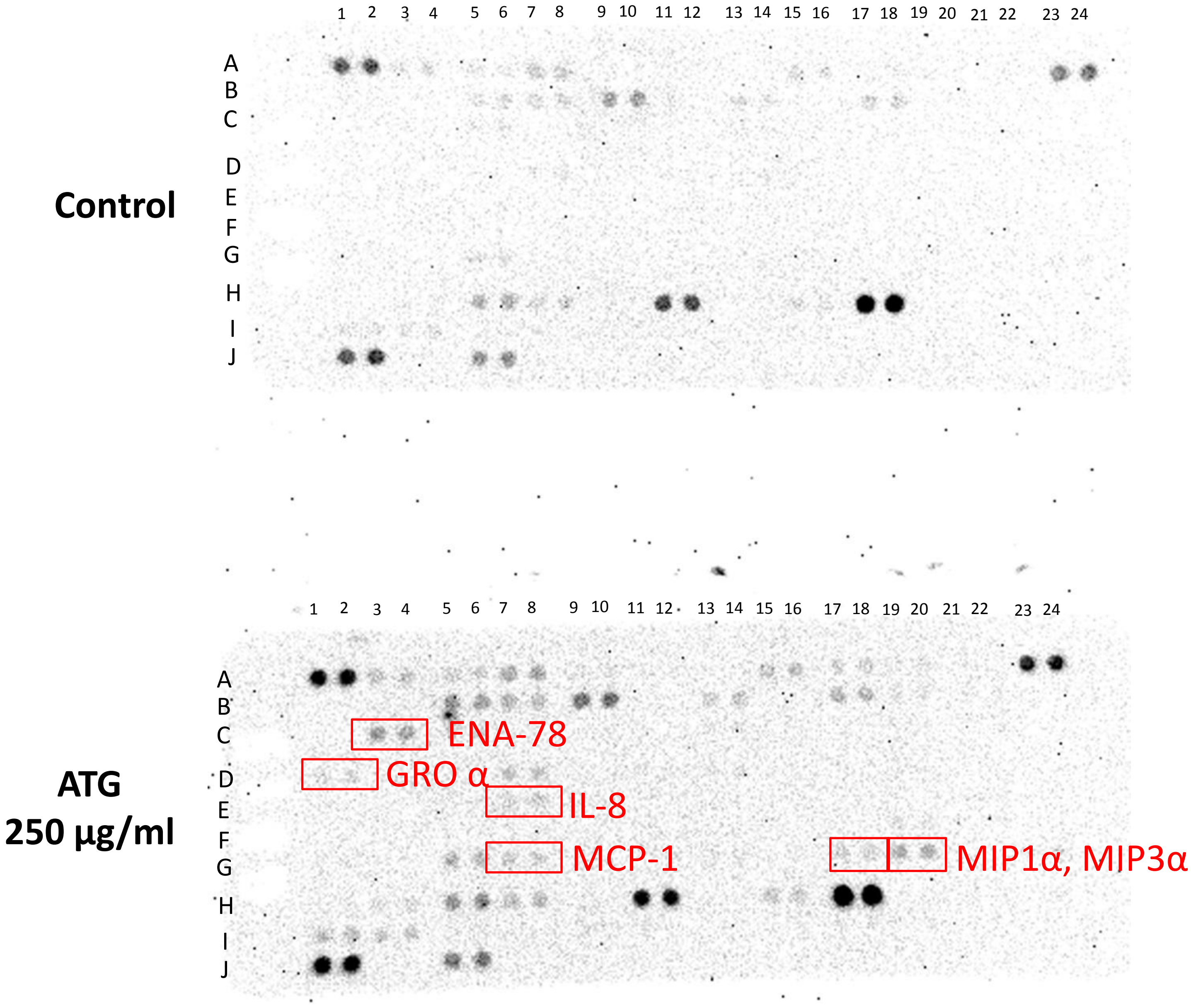

| Coordinate | Analyte | Coordinate | Analyte | Coordinate | Analyte |
|---|---|---|---|---|---|
| A1, A2 | Reference Spots | E1, E2 | IL-4 | H1, H2 | Myeloperoxidase |
| A3, A4 | Adiponectin | E3, E4 | IL-5 | H3, H4 | Osteopontin |
| A5, A6 | Apolipoprotein A-I | E5, E6 | IL-6 | H5, H6 | PDGF-AA |
| A7, A8 | Angiogenin | E7, E8 | IL-8 | H7, H8 | PDGF-AB/BB |
| A9, A10 | Angiopoietin-1 | E9, E10 | IL-10 | H9, H10 | Pentraxin 3 |
| A11, A12 | Angiopoietin-2 | E11, E12 | IL-11 | H11, H12 | PF4 |
| A13, A14 | BAFF | E13, E14 | IL-12 p70 | H13, H14 | RAGE |
| A15, A16 | BDNF | E15, E16 | IL-13 | H15, H16 | RANTES |
| A17, A18 | Complement Component C5/C5a | E17, E18 | IL-15 | H17, H18 | RBP-4 |
| A19, A20 | CD14 | E19, E20 | IL-16 | H19, H20 | Relaxin-2 |
| A21, A22 | CD30 | E21, E22 | IL-17A | H21, H22 | Resistin |
| A23, A24 | Reference Spots | E23, E24 | IL-18 Bpa | H23, H24 | SDF-1α |
| B3, B4 | CD40 ligand | F1, F2 | IL-19 | I1, I2 | Serpin E1 |
| B5, B6 | Chitinase 3-like 1 | F3, F4 | IL-22 | I3, I4 | SHBG |
| B7, B8 | Complement Factor D | F5, F6 | IL-23 | I5, I6 | ST2 |
| B9, B10 | C-Reactive Protein | F7, F8 | IL-24 | I7, I8 | TARC |
| B11, B12 | Cripto-1 | F9, F10 | IL-27 | I9, I10 | TFF3 |
| B13, B14 | Cystatin C | F11, F12 | IL-31 | I11, I12 | TfR |
| B15, B16 | Dkk-1 | F13, F14 | IL-32 | I13, I14 | TGF-α |
| B17, B18 | DPPIV | F15, F16 | IL-33 | I15, I16 | Thrombospondin-1 |
| B19, B20 | EGF | F17, F18 | IL-34 | I17, I18 | TNF-α |
| B21, B22 | Emmprin | F19, F20 | IP-10 | I19, I20 | uPAR |
| C3, C4 | ENA-78 | F21, F22 | I-TAC | I21, I22 | VEGF |
| C5, C6 | Endoglin | F23, F24 | Kallikrein 3 | J1, J2 | Reference Spots |
| C7, C8 | Fas Ligand | G1, G2 | Leptin | J5, J6 | Vitamin D BP |
| C9, C10 | FGF basic | G3, G4 | LIF | J7, J8 | CD31 |
| C11, C12 | FGF-7 | G5, G6 | Lipocalin-2 | J9, J10 | TIM-3 |
| C13, C14 | FGF-19 | G7, G8 | MCP-1 | J11, J12 | VCAM-1 |
| C15, C16 | Flt-3 Ligand | G9, G10 | MCP-3 | J23, J24 | Negative Controls |
| C17, C18 | G-CSF | G11, G12 | M-CSF | | |
| C19, C20 | GDF-15 | G13, G14 | MIF | | |
| C21, C22 | GM-CSF | G15, G16 | MIG | | |
| D1, D2 | GROα | G17, G18 | MIP-1α/MIP-1β | | |
| D3, D4 | Growth Hormone | G19, G20 | MIP-3α | | |
| D5, D6 | HGF | G21, G22 | MIP-3β | | |
| D7, D8 | ICAM-1 | G23, G24 | MMP-9 | | |
| D9, D10 | IFN-γ | | | | |
| D11, D12 | IGFBP-2 | | | | |
| D13, D14 | IGFBP-3 | | | | |
| D15, D16 | IL-1α | | | | |
| D17, D18 | IL-1β | | | | |
| D19, D20 | IL-1ra | | | | |
| D21, D22 | IL-2 | | | | |
| D23, D24 | IL-3 | | | | |
